# Detection and quantification of infectious severe acute respiratory coronavirus-2 in diverse clinical and environmental samples

**DOI:** 10.1038/s41598-022-09218-5

**Published:** 2022-03-30

**Authors:** Yi-Chan Lin, Rebecca J. Malott, Linda Ward, Linet Kiplagat, Kanti Pabbaraju, Kara Gill, Byron M. Berenger, Jia Hu, Kevin Fonseca, Ryan S. Noyce, Thomas Louie, David H. Evans, John M. Conly

**Affiliations:** 1 Department of Medical Microbiology and Immunology, University of Alberta, 6-142L Katz Group Centre, Edmonton, AB T6G 2J7 Canada; 2 Cumming School of Medicine, University of Calgary, 3030 Hospital Dr NW, Calgary, AB T2N 4W4 Canada; 3 Foothills Medical Centre, Alberta Health Services, 1403 29 Street NW, Calgary, AB 2TN 2T9 Canada; 4 Alberta Public Health Laboratory, Alberta Precision Laboratories, Calgary, AB Canada

**Keywords:** Infectious diseases, Microbiology, Infectious-disease diagnostics, Virology, Infectious diseases, Viral infection

## Abstract

To explore the potential modes of Severe Acute Respiratory Coronavirus-2 (SARS-CoV-2) transmission, we collected 535 diverse clinical and environmental samples from 75 infected hospitalized and community patients. Infectious SARS-CoV-2 with quantitative burdens varying from 5 plaque-forming units/mL (PFU/mL) up to 1.0 × 10^6^ PFU/mL was detected in 151/459 (33%) of the specimens assayed and up to 1.3 × 10^6^ PFU/mL on fomites with confirmation by plaque morphology, PCR, immunohistochemistry, and/or sequencing. Infectious virus in clinical and associated environmental samples correlated with time since symptom onset with no detection after 7–8 days in immunocompetent hosts and with N-gene based C_t_ values ≤ 25 significantly predictive of yielding plaques in culture. SARS-CoV-2 isolated from patient respiratory tract samples caused illness in a hamster model with a minimum infectious dose of ≤ 14 PFU. Together, our findings offer compelling evidence that large respiratory droplet and contact (direct and indirect i.e., fomites) are important modes of SARS-CoV-2 transmission.

## Introduction

Since February 2020, severe acute respiratory syndrome coronavirus-2 (SARS-CoV-2) has gripped the globe^1^. In response, public health measures were implemented based on the best available data related to the presumed modes of transmission and based on recommendations for other respiratory viruses^2–4^.

The modes for SARS-CoV-2 transmission are considered to occur through multiple routes including large respiratory droplets, contact (direct and indirect i.e. fomites), and small particle aerosols, with close contact being a major risk associated with transmission^5,6^. There has been debate about the degree to which respiratory secretions of varying particle sizes, including those produced by exhaled breath, may be responsible for transmission of the virus^5,7–10^ in part due to confusion over the relationships between a PCR signal and how that result relates to the underlying quantities of viral non-genomic RNA, virus genomes, and infectious virions. Transmission is further clouded by uncertainty over the minimal infectious dose in humans although classical human volunteer studies with the 229E coronavirus have shown clinically evident attack rates as high as 50% with extremely low inoculation doses of 0.6–1.5 TCID_50_^11,12^.

Recent reports suggest that there is little evidence to support transmission of SARS-CoV-2 through contaminated surfaces^13,14^ and the United States Centers for Disease Control and Prevention recently suggested that surfaces are not a significant mode of transmission of SARS-CoV-2^15^. However, extensive surface contamination with SARS-CoV-2 by a symptomatic patient has been demonstrated in a hospital setting^16^ where a link was established between the presence of environmental contamination and the quantity of SARS-CoV-2 RNA, using cycle threshold (C_t_), detected in the clinical sample, and day post-symptom onset and shedding of infectious SARS-CoV-2. Additional studies investigating shedding of infectious virus from COVID-19 patients consistently report that it is highest early in the course of infection^17–20^. It appears likely that patients early in the course of COVID-19 could more readily transmit and contaminate surfaces in the clinical and community setting, leading to an increased risk of virus transmission^21^. Our study was driven by the hypothesis that COVID-19 patients in the early stage of their illness would shed infectious SARS-CoV-2 in respiratory secretions and contaminate surfaces that can contribute to transmission of the virus. We conducted detailed virological assessments of infectious virus loads in COVID-19 patients at different stages of disease, assessing various clinical and environmental (fomite) samples taken from the hospital and community setting, to gain a further understanding of the potential modes of SARS-CoV-2 transmission and to explore the reasons why this virus is so contagious.

## Results

### Patient characteristics and symptoms

Details of the infected cases are provided in Table 1. All (41/41; 100%) of the inpatient cases were found to have symptoms and/or signs consistent with COVID-19 using comprehensive data gathering and attention to reducing any potential biases. Of the community cases, the vast majority (32/34; 94.1%) were also found to exhibit symptoms and signs compatible with COVID-19. Of the total of 75 persons with COVID-19, 86.7% (65) had one or more “core respiratory” symptoms/signs including cough, sore throat, dysphagia, nasal congestion or rhinorrhea, dyspnea, or difficulty breathing at some point during their illness. The remainder had “other” non-respiratory symptoms (e.g., fever, nausea, emesis, fatigue, fever, muscle aching, dys[a]geusia and/or dys[an]osmia) which have been associated with COVID-19. We identified only 2 persons (2.7%) with complete absence of symptoms in our dataset.

**Table 1:** Clinical characteristics of COVID-19 cases (n = 75).

Basic demographics	N (%)
Age (years), mean ± SD (range)	53.3 ± 10.6 (1 d to 90 yr)
Female	41 (54.7)
Direct admission	31 (41.9)
Admitted from care facility	6 (8.1)
Admitted from transition housing	4 (5.4)
Community (not admitted)	34 (45.3)

Co-morbidities*	N
Cardiac disease and/or hypertension	27
Metabolic disorders (other than diabetes)	27
Musculoskeletal diseases	18
Respiratory	15
Diabetes (Types 1 and 2)	13
Malignancy	13
Digestive disorder	13
Mental health	11
Skin and soft tissue disorders	11
Obesity	10
Infections (non-respiratory)	8
Renal disease	6
Alcohol/substance use disorder	6
Peripheral vascular disease	5
Solid organ transplant (< 3 months post-transplant)	2
Other	4
No underlying co-morbidities^†^	10

Presenting symptoms and signs^‡^	N (%)
Asymptomatic	2 (2.7)
Symptomatic	73 (97.3)
Cough (productive or non-productive)	38 (50.6)
Fatigue/exhaustion	33 (44.0)
Fever/sweating/chills	31 (41.3)
Shortness of breath/difficulty breathing	29 (38.7)
Muscle or joint ache or pain	28 (37.3)
Sore throat/painful swallowing	27 (36.0)
Headache	16 (21.3)
Loss of/change to sense of smell or taste	15 (20.0)
Runny nose/nasal congestion	15 (20.0)
Emesis/nausea/loss of appetite	8 (10.7)
Altered mental status	8 (10.7)
Chest pain/crackles/congestion	7 (9.3)

*Co-morbidities are not mutually exclusive.

^†^Mostly community participants.

^‡^All symptoms and signs (mean of 3.5 per patient).

The symptom complexes had a variable presentation, with new symptoms/signs developing over the course of the illness while others settled but 88% (64/73) of those with identified symptoms and/or signs had at least three identified symptoms/signs. We identified 3 (4%) presymptomatic persons where symptoms and/or signs followed within 24–72 h after identification of a positive PCR test.

Core respiratory symptoms (any one of or a combination of cough, sore throat, nasal congestion/rhinorrhea, and dyspnea) were found at some point in the illness course in just over 85% of our 75 person cohort and almost 90% had at least 3 identified symptoms/signs compatible with an expanded list of COVID-19 compatible symptoms and signs.

### Detection of infectious SARS-CoV-2 in samples from COVID-19 patients and their environment is associated with day post-symptom onset

**Figure 1:**
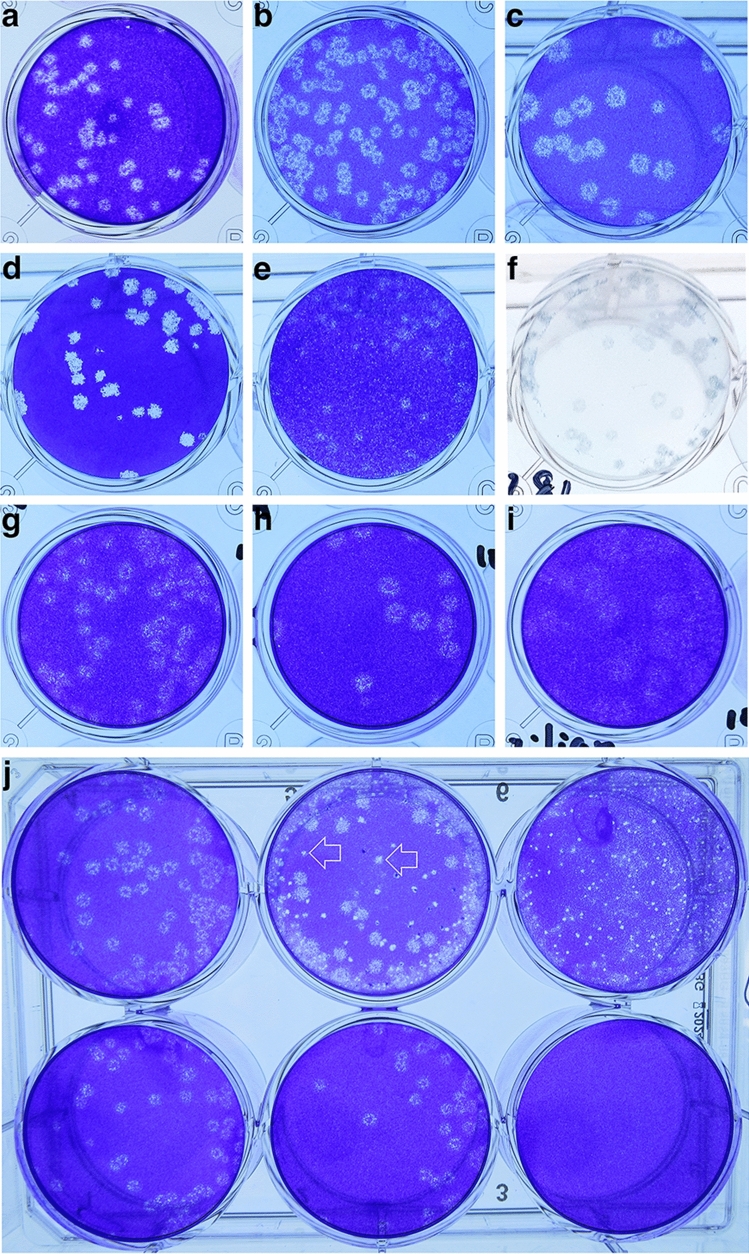
SARS-CoV-2 plaque morphology. (**a–c**) Variation in plaque sizes when clinical samples are plated on Vero CCL-81 cells. Note the characteristic halo structure. (**d**) SARS-CoV-2 plaques on Vero E6/TMPRSS2 cells. (**e–f**) SARS-CoV-2 plaques on CCL-81 cells stained with crystal violet (**e**) and immunohistochemical staining with an anti-SARS-CoV-2 spike antibody (**f**). (**g–i**) SARS-CoV-2 variants of concern on Vero CCL81 cells (**g**: Alpha; **h**: Beta; **i**: Gamma). **j** Patient samples passed through 0.2 mm filters to remove bacterial and fungal contamination. Top row: unfiltered samples; Bottom row: filtered samples. Arrows show examples of false plaques caused by growth of bacteria/fungi on the cell monolayers. Figure prepared using Photoshop v23.0 (https://www.adobe.com).

A diagnostically rich sampling strategy was used to collect multiple clinical and associated environmental sample types from COVID-19 patients at different times post-symptom onset. SARS-CoV-2 causes a distinct cytopathogenic effect (CPE), growing quickly in culture and producing large, haloed plaques in the Vero CCL-81 cell monolayer in 2–3 days (Fig. 1). Variable sized plaques were formed by viruses isolated from different patients which were attributed to different mutations in the viruses (Fig. 1a). A few specimens exhibited atypical plaques and on close inspection these were produced either by other viruses (e.g., Herpes simplex virus) or were artifacts caused by the destruction of the monolayer by bacteria or fungi (Fig. 1b). For example, a specimen collected from an immunosuppressed patient with oral candidiasis, had to be passed through a 0.2 µm filter and cultured with additional anti-mycotic drugs to titer the SARS-CoV-2. Occasionally virus with typical plaques was cultured from samples with low C_t_ values after more prolonged incubation and these samples were re-titered on Vero E6/TMPRSS2 cells to ensure accurate quantification^22^. In two epidemiologically-linked cases the viruses formed plaques that differed in appearance from those formed on Vero CCL-81 cells (Fig. 1a). In such cases, immunohistochemistry was used to confirm the identity of the isolated virus as SARS-CoV-2 (Fig. 1a). Plaque counts varied across the many different samples, ranging from near the limits of detection (~ 5 PFU/mL) to > 10^6^ PFU/mL.

**Figure 2:**
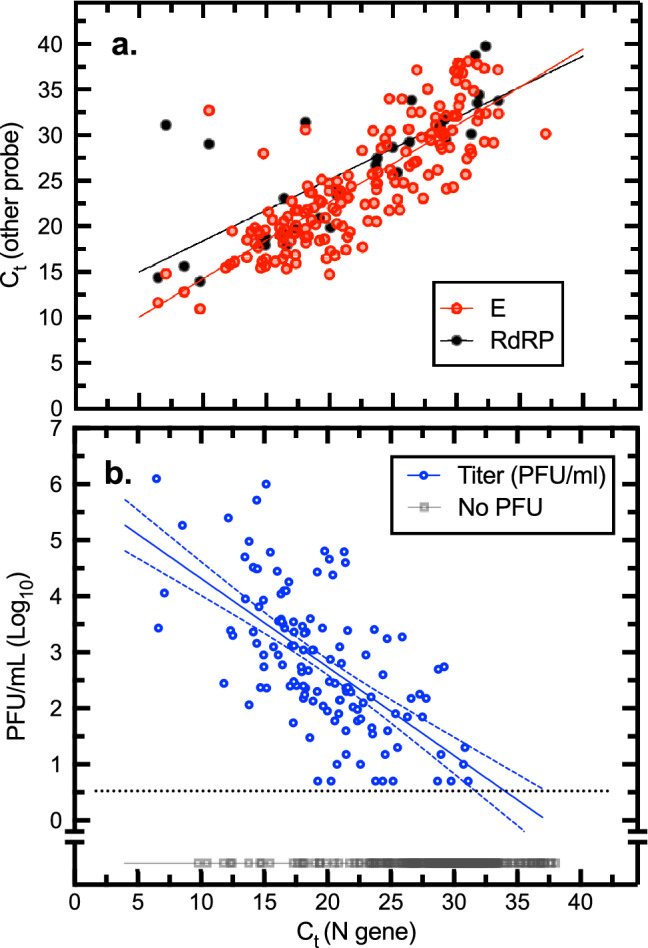
SARS-CoV-2 detection using PCR and plaque assays. (**a**) Comparison of reverse transcriptase qPCR assays. At the mid-point of the plot, the N gene-based assay generates C_t_ values that are about two values lower that C_t_ measured using E or RDRP gene primers. The solid lines were calculated from a linear regression (y = 0.84x + 5.9, goodness of fit r^2^ = 0.67; y = 0.68 + 11.6, goodness of fit r^2^ = 0.59) for E and RdRP assays, respectively. (**b**) Relationships between N gene-based C_t_ values and virus titer measured as plaque forming units (PFU). The samples exhibited a wide range of virus titers varying from > 10^6^ PFU/mL to the limit of detection (~ 5 PFU/mL). A linear regression fitted to the log_10_-transformed data is also shown (y =  − 0.16x + 5.9; r^2^ = 0.41) along with the 95% confidence intervals. Specimens bearing no detectable infectious SARS-CoV-2 virus are also plotted for purposes of comparison (black squares). Most of the specimens (97%) were titered on Vero CCL-81 cells. Figure prepared using Prism v9.3 (https://www.graphpad.com).

SARS-CoV-2 from each sample was also detected by RT-PCR using primer sets targeting the E and RdRP genes. A comparison of all these molecular data is shown in Fig. 2a. There was a positive correlation between the C_t_ measured using the three SARS-CoV-2-specific probes (E versus N2, Pearson correlation r = 0.82, *P* < 0.0001; RdRP versus N2, r = 0.77, *P* < 0.0001). N2 gene probes generated lower C_t_ values than E and RdRP probes, with the corresponding C_t_ being 1.7–2.4 C_t_ values higher for E and RdRP probes, respectively.

Overall, 33% (142/429) of the plated specimens exhibited some quantity of infectious virus, with considerable scatter in the titers**,** and some clinical specimens contained substantial amounts of virus (Fig. 2b). At the high end of the infectious spectrum, a blood-tinged sample of sputum was retrieved from a cotton gown a few minutes after being coughed up and found to have a C_t_ of 6.47 (N gene) and a titer of 1.3 × 10^6^ PFU/ mL. This titer is comparable to that which can be obtained when SARS-CoV-2 is cultured on Vero cells and their related derivatives (e.g., Vero E6/TMPRSS-2 cells), where we routinely obtain titers ranging from 10^6^ to 10^7^ PFU/mL. Another specimen acquired from an endotracheal tube had a titer of 1.0 × 10^6^ PFU/mL and the many other specimens with titers in the 10^4^-to-10^5^ PFU/mL range show that that this level of viral load is likely common, illustrating the high quantitative burden which SARS-CoV-2 can achieve in the human respiratory tract.

**Figure 3:**
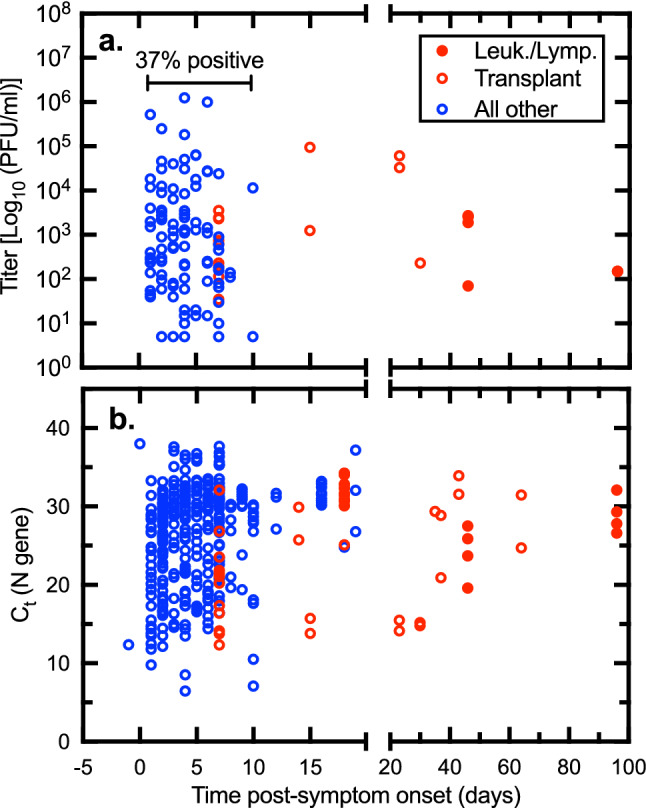
Impact of sample timing on SARS-CoV-2 virus detection. The time post-onset was calculated from interviews and/or chart review. (**a**) Virus titer where it could be detected. (**b**) All the C_t_ measurements acquired over the study. In most cases, the capacity to detect virus drops off precipitously about a week after case onset (blue data points). However, both RNA (lower panel) and PFU (upper panel) are detected for many days or weeks later where the patient is immunocompromised (red data points). Figure prepared using Prism v9.3 (https://www.graphpad.com).

Specimens were obtained from patients on different dates post-symptom onset, ranging from 1 to > 90 days. In this regard the date of onset was evaluated using both chart reviews and interviews. The infectious specimens were typically detected in the first week after symptom onset (Fig. 3a). Within a window extending up to 8-days post onset, 37% (114/308) of specimens contained infectious virus (Fig. 3a). Thereafter, the C_t_ values rose to levels unlikely to yield plaques and indeed, no plaques were found. We continued to detect viral nucleic acids for another 2 or more weeks in some patients (Fig. 3b). The exceptions to this trend were seen in patients characterized by some type of immunodeficiency. Two solid organ transplant patients with a persistent carriage of infectious virus eventually responded to Remdesivir treatment combined with a reduction in their immunosuppressive regimens^23^. We also collected specimens from a patient with follicular lymphoma who had infectious virus from saliva and NP swabs which were plaque positive (1.5 × 10^2^ PFU/mL) 5 months from the date of original symptom onset (Fig. 3a,b).

### Quantitative burden of infectious SARS-CoV-2 by sample type

**Figure 4:**
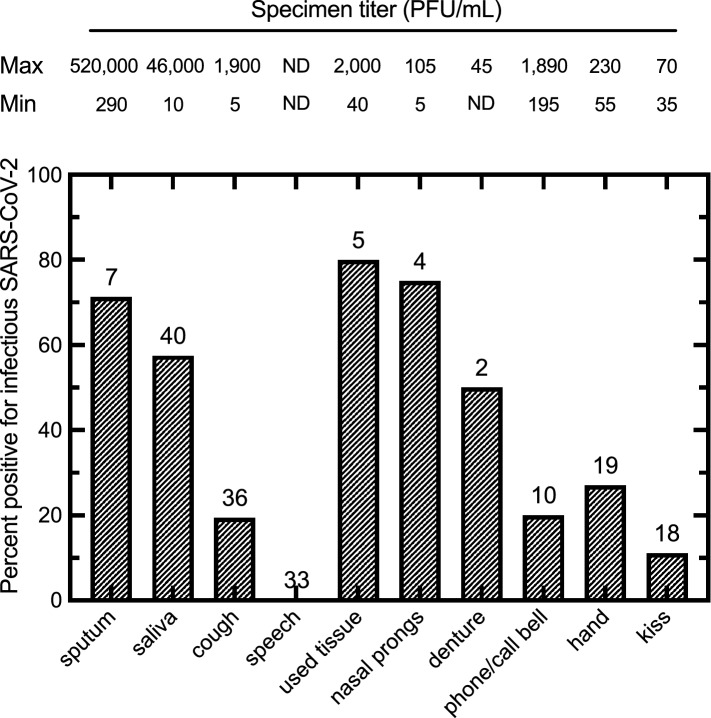
Percentage of clinical and environmental samples positive for infectious SARS-CoV-2 from patients with positive NP or TS by infectious titer. The number of samples acquired in each category are indicated above each bar, along with the minimum and maximum infectious titer (PFU/mL) observed for each sample along the top of the figure. Not all sample types were collected from every patient. Sputum indicates productive cough samples. ND = None detected. Figure prepared using Prism v9.3 (https://www.graphpad.com).

Of the samples from patients with concomitant nasopharyngeal (NP) or throat swab (TS) specimens positive for infectious SARS-CoV-2, the expectorated sputum samples had the highest percentage of positive samples and highest virus titers (71%, 2.9 × 10^2^ to 5.2 × 10^5^ PFU/mL), with saliva being the next most positive sample type (58%, 1 × 10^1^ to 4.6 × 10^4^ PFU/mL), followed by cough samples without discernible sputum (19%, 5 × 10^0^ to 1.9 × 10^3^ PFU/mL) (Fig. 4). The presence of infectious virus with high quantitative burdens was found in 28% of all productive and non-productive cough specimens, which would have contained droplet particles of many different sizes. Some patients who did not have cough as a part of their symptom complex were asked to produce a cough which was not as natural as an illness-associated cough which may have affected the positivity result. No infectious virus was recovered from 33 continuous speech samples with a known NP or throat swab positive for infectious virus (Fig. 4). We also detected infectious SARS-CoV-2 in samples acquired from random washes of patients’ hands with culture media (28%, 6 × 10^1^ to 2.3 × 10^2^ PFU/mL) and two kiss samples were found to be positive as well (11%, 3.5 × 10^1^ and 7.0 × 10^1^ PFU/mL) (Fig. 4). We found one tissue specimen from a placenta with infectious virus at a titer of 2.8 × 10^2^ PFU/mL from a mother who had had active COVID-19 infection at the time of delivery.

The environmental samples that were most commonly found to contain infectious virus were used facial tissues (80%, 4.0 × 10^1^ to 2.0 × 10^3^ PFU/mL), nasal prongs (75%, 5 × 10^0^ to 1.1 × 10^2^ PFU/mL), and dentures found at the bedside which had lain there for approximately 4 h (50%, 45 PFU/mL) (Fig. 4). Some phone and call bell samples were found to contain infectious virus (20%, 2.0 × 10^2^ to 1.9 × 10^3^ PFU/mL) (Fig. 4), although no data were collected on the frequency of cleaning of these items. Other miscellaneous environmental samples were collected over the evaluation period included a used facecloth found lying on the bedsheets with 1.2 × 10^2^ PFU/mL and a sputum sample deposited on a bedrail and allowed to dry for 30 min had an infectious titer found of 1.5 × 10^3^ PFU/mL. No infectious virus was detected on either of two pulse oximeters sampled from patient rooms.

### Hand to hand transfer of SARS-CoV-2

A patient who was 1 day post symptom onset with a NP swab C_t_ of 18.6 was found to have a throat swab virus titer of 4.0 × 10^3^ PFU/mL and a cough sample titer of 5.2 × 10^5^ PFU/mL. After coughing onto their right hand and then shaking their cleansed left hand, both the primary inoculation hand and the receiving hand samples were positive for infectious virus with similar findings of 1.4 × 10^2^ and 3.0 × 10^2^ PFU/mL, respectively. Although only a single experiment, the result illustrates the capacity to transfer the virus from hand to hand.

### Effects of drying on the titer of SARS-CoV-2 in clinical samples of saliva

**Figure 5:**
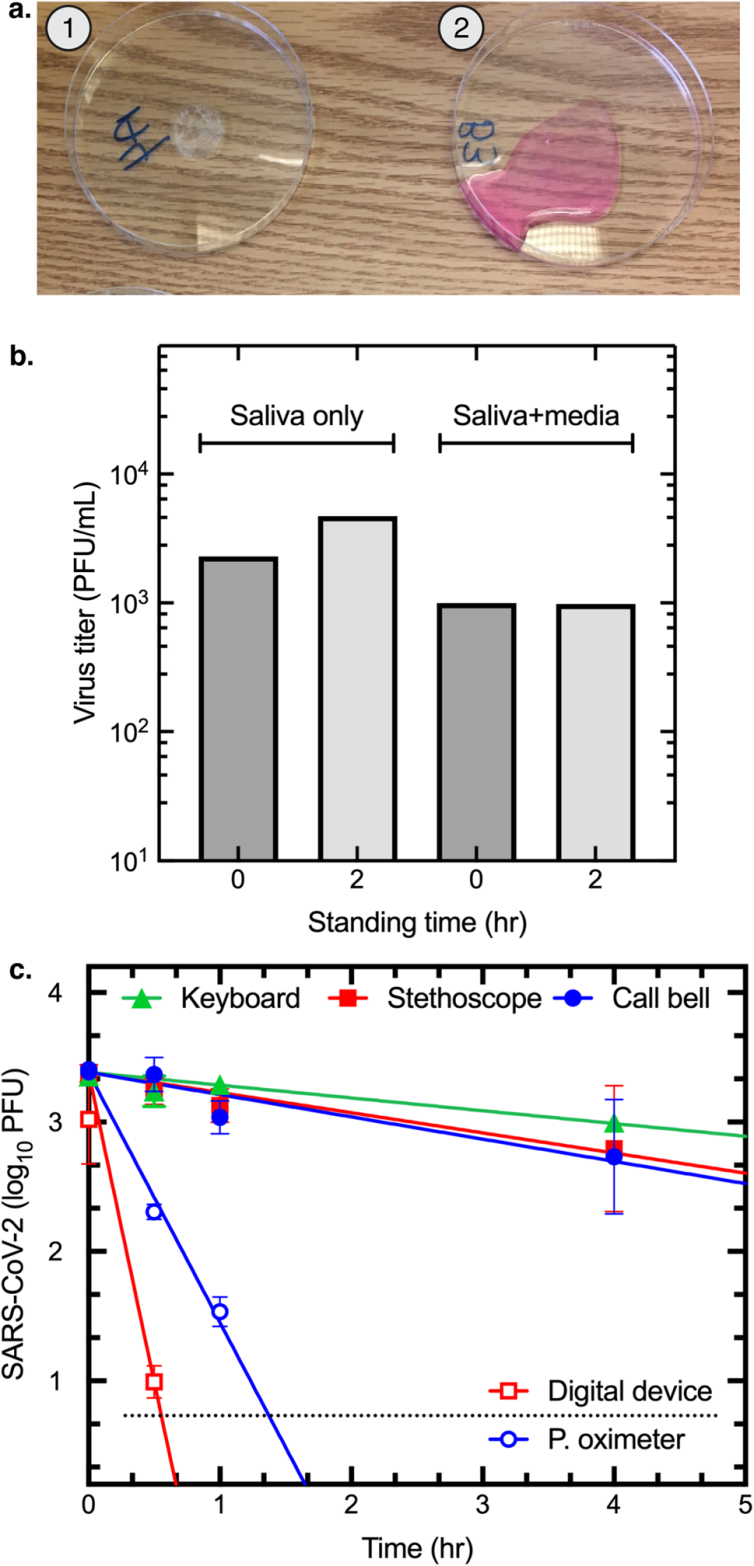
Stability of SARS-CoV-2. (**a**) Saliva from a COVID-19 patient, or saliva mixed with DMEM + serum, were left in open Petri dishes in a patient care room. Image showing the effects of leaving a saliva sample (dish 1), or saliva mixed with DMEM + serum (dish 2) for 2 h. The saliva specimen dried completely. (**b**) Effect of standing time and drying on virus titers. The two control samples (blue bars) were stored on ice in closed tubes during the two-hour experiment. (**c**) Representative patient-contacted surfaces were acquired from the patient care setting and transferred for testing without further treatment beyond everyday maintenance and cleaning. Some were partly disassembled to facilitate safe handling and access. An endotracheal tube sample, containing 1 × 10^6^ PFU/mL SARS-CoV-2 diluted in DMEM, was applied in three 10 µL volumes to each item and either retrieved immediately, or stored in a biocontainment hood for the indicated times before recovery and plaque assay. The figure shows a linear regression applied to the log_10_-transformed plaque counts. The half-lives were separately calculated from a non-linear fit to the untransformed data (not shown) and ranged from 3 min (digital device cover) to 82 min (keyboard). Figure assembled using Illustrator v26.0 (https://www.adobe.com) and Prism v9.3 (https://www.graphpad.com).

We examined the stability of SARS-CoV-2 in dried saliva in a patient care setting. COVID-19 patient saliva samples were allowed to dry over 2 h on an uncovered sterile plastic surface at room temperature (Fig. 5a). After being resuspended in DMEM + , drying had essentially no effect on the virus titer relative to that measured in samples that had not been dried, or samples that were first diluted in DMEM + and stored at room temperature for 2 h. All the titers exceeded ≥ 10^3^ PFU/mL in the different control and experimental specimens (Fig. 5b). As a further illustration of the stability of SARS-CoV-2 in the patient care setting in dried secretions, infectious virus was recovered from a used facial tissue that had been overlooked on a side table in a COVID-19 patient care room for 9 h (40 PFU/mL on TMPRSS2 cells).

### Virus stability on clinical equipment

We examined the stability of SARS-CoV-2 on items that are routinely used in a patient care setting (Fig. 5). At time 0, there was no difference in the amount of virus recovered from the stethoscope diaphragm, pulse oximeter, bedside call bell, and keyboard. However, the amount of virus eluted from a personal digital device cover was significantly lower than the other four medical item samples (ANOVA test, *p* = 0.0495). Presumably this is because the materials of the cover hinder recovery of the virus or there were residual virucidal chemicals present on the surface. Infectious virus was recovered from the call bell, computer keyboard, and stethoscope diaphragm for up to 4 h; for at least one hour on the pulse oximeter and 30 min from the personal digital device cover (Fig. 5). We also spotted 10 µL sputum, each containing 1.2 × 10^3^ PFU of SARS-CoV-2, onto coupons cut from a N95 respirator to mimic a COVID-19 patient coughing and depositing saliva or sputum on the outer surface of a healthcare worker’s personal protective equipment. After drying in room air for 1 h, an average of 6.7 × 10^2^ PFU (55%) of the virus was recovered from each coupon, demonstrating that SARS-CoV-2 retains infectivity in dried sputum on the outer surface of a N95 respirator.

**Figure 6:**
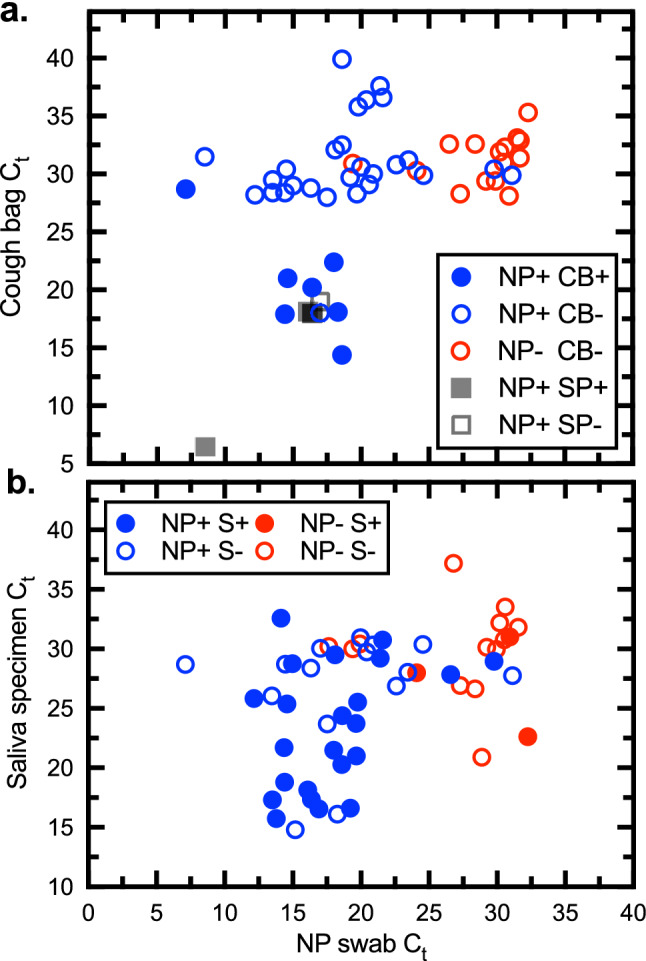
Infectious SARS-CoV-2 in saliva, sputum, and cough specimens. (**a**) Virus samples were acquired from NP swabs as well as cough bag samples (CB), sputum (SP), or saliva (S). Samples determined to contain infectious virus are indicated with solid-colour coding. (**b**) Virus samples were acquired from NP swabs as well as saliva. A C_t_ ≤ 25 in the NP swab predicts that about 1/3–1/2 of the cough/sputum and saliva specimens will also bear infectious virus, respectively. Figure prepared using Prism v9.3 (https://www.graphpad.com).

We further examined samples of cough droplets (i.e., macroscopically visible droplets) captured in transparent polyethylene bags, to examine the relationship between C_t_ values measured in NP swabs and cough samples (Fig. 6a). Each datum point was further color-coded to show the viral culture quantitative burden. Patients with a high virus load (low NP C_t_ values) and early in the course of their illness (5.5 ± 2.9 days post-onset, n = 8) produced droplets with high titers of infectious virus. Cough droplets and sputum arise from both the upper and lower respiratory tract, although sputum generally represents a larger, more semi-solid respiratory-type secretion and is more easily collected. Most of the sputum specimens exhibited low C_t_ values (< 20) (Fig. 6a) and 71% had infectious virus (Figs. 4 and 6a). An analysis of saliva specimens showed a similar pattern (Figs. 4 and 6b), although in contrast to the cough/sputum samples a low C_t_ in the NP specimen was not always predictive of whether virus would be detected in saliva samples (Fig. 6b).

### Correlation of C_t_ (N gene) with infectious virus titers

**Figure 7:**
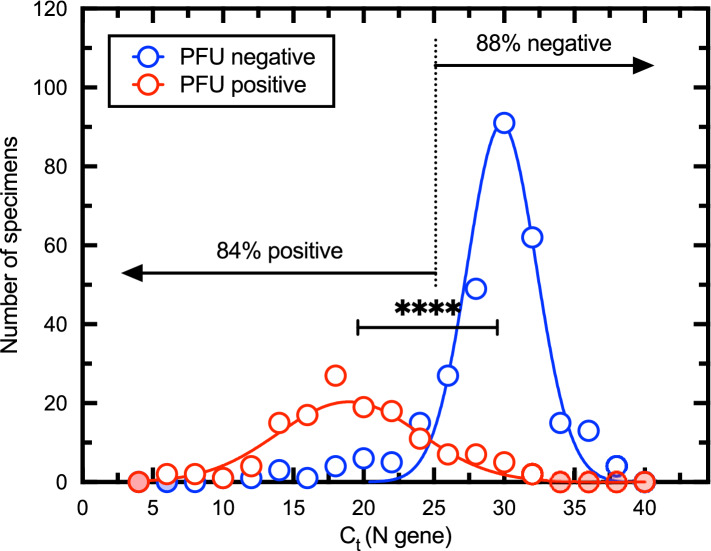
Relationship between C_t_ and positive plaque assays. The specimens were divided into plaque-positive, and plaque-negative categories and the distribution of C_t_ values calculated using bin steps of two. A non-linear fit of two Gaussian curves to these data is also shown. A sample with a C_t _≤ 25 is 84% likely to bear infectious material while samples with C_t_ > 25 are 88% negative. An unpaired t-test, using Welch’s correction for unequal variances and sample sizes, indicates that the two means (19.6 ± 5.1 SD versus 29.2 ± 4.2 SD) are significantly different (two-tailed *P* < 0.0001). Red or blue filled shading indicates that the binning process counted no specimens in these bins. Figure prepared using Prism v9.3 (https://www.graphpad.com).

To determine how well C_t_ is predictive of infectious titer, samples that contained infectious material exhibited significantly (*P* < 0.0001) lower C_t_ values compared with plaque negative specimens (Fig. 7). Infectious vs. non-infectious samples exhibited a mean C_t_ of 19.6 ± 5.1 SD and 29.2 ± 4.2 SD, respectively. These data showed that virus plaques are difficult to recover from samples exhibiting a C_t_ > 25 and even when recovered (n = 18), the titers were low (median 7 × 10^1^ PFU/mL). Three samples had a C_t_ at 30 or 31 and were just at or slightly above the threshold of detection of 5 pfu/ml. Below a C_t_ of ≤ 25 (N gene assay) 84% of all samples we studied contained infectious virus. This C_t_ value offers a convenient benchmark that may be useful when evaluating the potential transmission risk posed by clinical and environmental specimens.

### SARS-CoV-2 RNA to PFU ratio

**Figure 8:**
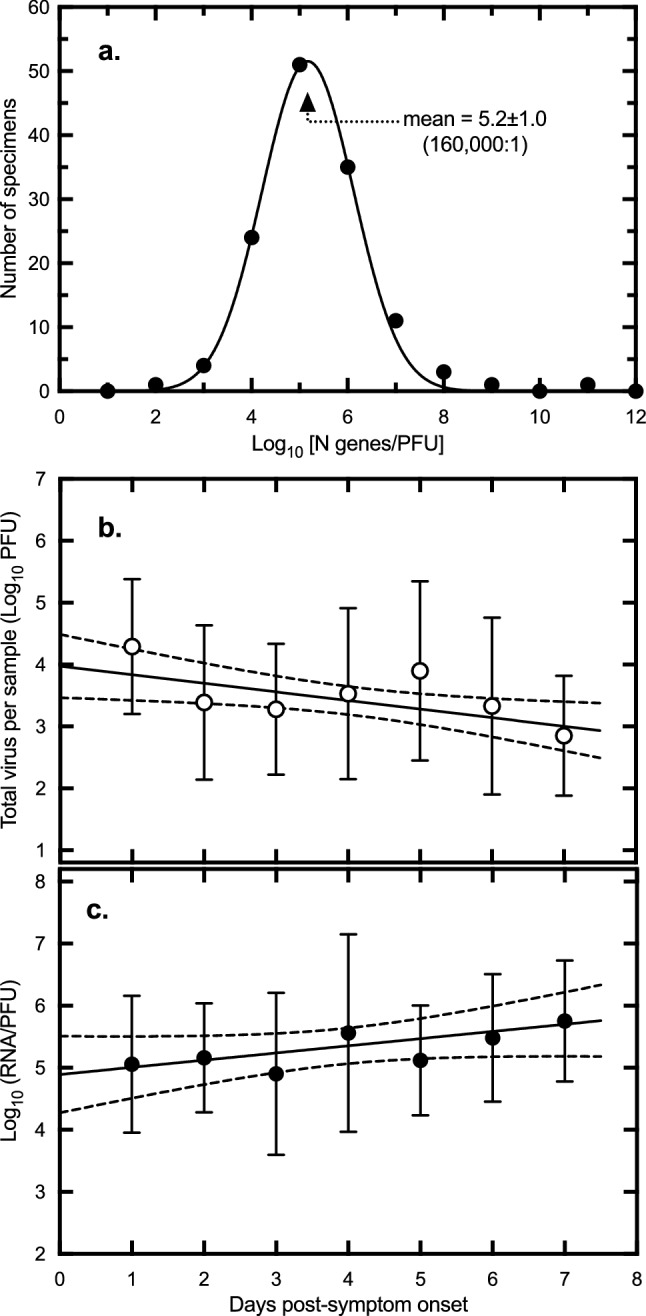
Relationship between viral RNA quantity, PFU, and sample timing. (**a**) The ratio of RNA to PFU was calculated using the virus titer plus a determination of the number of N-gene copies across all of the specimens. The ratios were log_10_ transformed and the distribution calculated across bin steps of one log_10_. A non-linear fit of a Gaussian curve to these data is also shown, centered on a mean of 5.2 ± 1.0 SD. This represents 10^5.2^ = 160,000 RNA copies per PFU. (**b**) The total quantity of virus in each infectious specimen was calculated using the titer (PFU/mL) and the known collection volumes (i.e., specimen + carrier/diluent). These values were then averaged across all of the samples for each of the indicated days. The plot shows a linear regression fitted to the log_10_-transformed data along with the 95% confidence intervals (y =  − 0.14x + 4.0, goodness of fit r^2^ = 0.05). The negative slope is significantly non-zero (*P* = 0.015, F-test). Error bars represent standard deviation. The average virus load declined about tenfold over the course of a week beginning at ~ 10^4.0^ PFU/mL on a hypothetical day zero. (**c**) The relative infectivity was calculated using the virus titer (PFU/mL) and specific quantity of virus RNA/mL in each sample. The plot shows a linear regression fitted to the log_10_-transformed data along with the 95% confidence intervals (y = 0.12x + 4.9; goodness of fit r^2^ = 0.04). The positive slope is significantly non-zero (*P* = 0.032, F-test). Error bars represent standard deviation. The ratio of RNA/PFU increased about eightfold over the same seven days, starting at ~ 10^4.9^ RNA/PFU on day zero. Figure prepared using Prism v9.3 (https://www.graphpad.com).

Another striking feature of the data, which has been previously reported by others^24^, is the very high ratio of viral RNA to PFU. This finding leads to misunderstandings regarding the health risk posed by samples with high C_t_ values. To calculate this parameter, all the qPCR assays incorporated additional wells containing known quantities of the N gene template. This allows one to estimate the starting quantity (SQ) of viral target sequences by comparing C_t_ values. This data, combined with the titer and known volumes of materials assayed, permits an estimate of the RNA/PFU ratio. Figure 8a illustrates this point where we have calculated the number of RNA copies (from the SQ/mL data) per plaque forming unit (from the PFU/mL). We observed a Gaussian distribution of values centered on a mean of 10^5.2 ± 1.0^ (i.e., 160,000) RNA targets per PFU. The method is not ideal as it makes assumptions about the efficiency of RNA extraction, PCR amplification, and plating. However, it isn’t greatly different from ratios calculated using the more homogeneous virus that can be harvested from culture (~ 10,000:1, data not shown).

Figure 8a was compiled from all the infectious specimens acquired over a range of days post-infection and this led us to wonder whether the timing of sample collection might have had an impact on the RNA/PFU ratios. To do this we focused on the specimens collected in the seven-day window post-symptom onset (Fig. 3), but still comprising a variety of specimen types. We observed that the calculated average of the total quantity of virus detected in each of the specimens declined about ninefold over the week after symptom onset from 9.5 × 10^3^ to 1.1 × 10^3^ PFU (Fig. 8b). At the same time the average RNA/PFU ratio rose ~ sixfold from 0.78 × 10^5^ to 5.0 × 10^5^ target copies/PFU (Fig. 8c). For practical reasons it is difficult to sample on the day of symptom onset but extrapolating these plots back to a hypothetical day zero shows that the highest virus loads (PFU) and most infectious virus (RNA/PFU) would be found at that time point.

### Virulence studies in a Syrian hamster model

**Figure 9:**
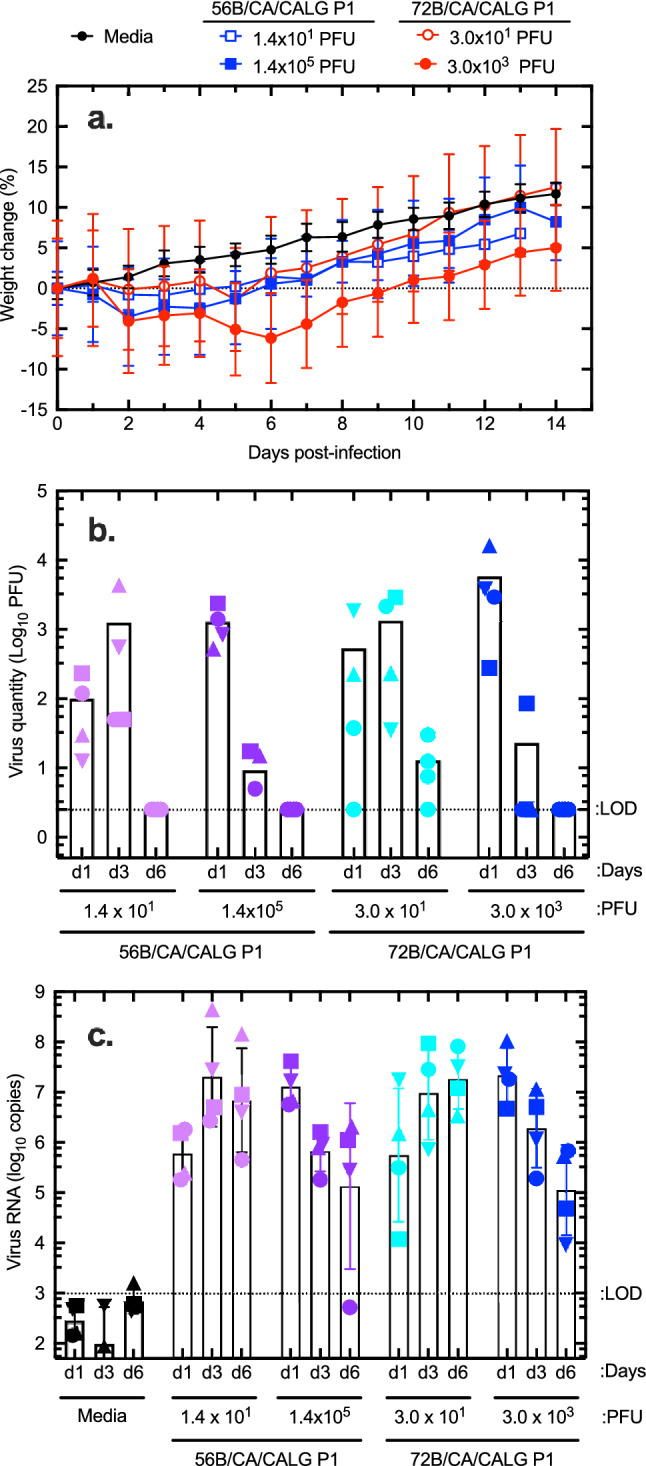
Virulence testing in Syrian hamsters. Two of the virus specimens (56B and 72B) were plaque purified and expanded to higher titers with one passage. The two stocks were then used to inoculate four groups of hamsters (4 per group) with the indicated doses of virus. Four control animals were also inoculated by the same intranasal route, with an equal volume (100 µL total) of serum-free media. (**a**) Shows the weight change relative to the starting weight for the animals in each of the five groups. Error bars represent standard error of the mean. A nasal swab was collected from each animal on days 1, 3 and 6, post-inoculation, and assayed for virus by plaque assay and virus RNA by qPCR (**b** and **c**). Both virus specimens produced weight loss and high titers of intranasal virus were detected at days 1 and 3 post-infection. Characteristically the lower doses (14 PFU for 56B and 30 PFU for 72B) yielded the most virus on day 3 post-infection, whereas the higher doses induced the highest levels of infection immediately after challenge, on day 1. The RNA is more persistent than virus, and it could still be detected 6 days post-infection in all of the infected animals, whereas no virus could be detected at this date. The animals were euthanized at day 14. The dashed lines show the limits of virus and RNA detection (LOD) in nasal swabs. Figure prepared using Prism v9.3 (https://www.graphpad.com).

Syrian golden hamsters are highly susceptible to SARS-CoV-2^25^. To document the in vivo infectivity of these clinical specimens, two different virus isolates were plaque purified from respiratory tract samples and expanded once on Vero cells. Genome sequencing showed that these belonged to the B.1.279 and B.1.128 lineages widely circulating in Alberta at the time. They were then used to inoculate hamsters by the intranasal route. Figure 9 shows the results of this experiment. Both isolates were highly infectious in hamsters, with a dose as low as 14 PFU causing the transient weight loss that characterizes this model (Fig. 9a). Nasal swabs were collected at days 1, 3, and 6 post-infection and detected virus replication that reached titers as high as 10^4^ PFU per swab, far more than the input doses. By day six post-infection, infectious virus could no longer be detected (Fig. 9b). No plaques were recovered from the lung homogenates at the end of the experiment (day 14) although some residual RNA was still detectable (not shown). Typically, the peak of infection was delayed a few days with the two lowest doses of virus, it was three days post-infection with infectious doses of 14 or 30 PFU, but just one day with the two highest doses. The levels of virus RNA paralleled the virus titers (Fig. 9c). We also calculated the ratio of RNA to PFU in samples acquired on days 1 and 3, which ranged from 4000 to 100,000 RNA/PFU in different animals and were ~ fivefold higher on day 3 (average 53 × 10^3^; range 13 × 10^3^ to 100 × 10^3^ RNA/PFU) than on day 1 (average 11 × 10^3^; range 4.0 × 10^3^ to 20 × 10^3^ RNA/PFU). Unfortunately, the small sample size precludes drawing further conclusions from the study. However, it is apparent that these low-passage SARS-CoV-2 specimens are highly infectious even at low doses and exhibit the same high ratios of RNA to PFU detected in human clinical specimens.

## Discussion

Our data demonstrate that the timing of collection of clinical specimens is of great importance relative to recovering infectious virus which could be detected for an average of 4–5 days after symptom onset but declined dramatically 7–8 days after symptom onset in both immunocompetent community and hospitalized inpatients. Selecting patients early in the course of their illness with lower C_t_ values confirmed our hypothesis that this diagnostic rich sampling strategy maximized the likelihood of detecting infectious virus and illustrates the relatively narrow window of infectivity beyond which one is unlikely to recover infectious virus. This finding is consistent with other studies which have reported that infectious virus is shed at highest levels from SARS-CoV-2 infected persons early in the course of infection^17,18,20,26–28^. Our findings also illustrate the risk posed to others regarding the maximal transmissibility period by most COVID-19 patients and serve to underscore the importance of potentially prolonged transmissibility in those with immunodeficiency syndromes which has been reported in other literature^29,30^.

Consistent with the suggestions on systematic symptom assessment and serial follow up^31^, our comprehensive interviewing strategy and serial follow up found that over 97% of all patients had symptoms and/or signs although our dataset was predominantly adults with only 2 pediatric cases. In elderly cognitively impaired persons who would not be able to provide reliable interview responses, signs such as rhinorrhea, an elevated temperature, tachypnea or sputum production were noted by experienced health care workers which added to the precision of the findings. Multiplicity of symptoms and signs was frequent, and serial follow up and secondary interviews over time were extremely useful. Our findings are congruent with a recent systematic review^32^ which used a fixed effect meta-analysis and found asymptomatic cases represented only 17% (95% CI 14% to 20%) of COVID-19 cases. A recent study of household transmission where a comprehensive capture of symptoms was done with use of a daily symptom monitoring tool, review of classic and non-classic symptoms plus initially daily RT-PCR testing, found 100% (12/12) of COVID-19 patients were symptomatic and is consistent with our data^33^.

We found a strong correlation between the E and N gene C_t_ values and that a C_t_ ≤ 25 in the N gene assay was strongly correlated with the likelihood of finding infectious virus in both clinical and environmental samples. We found infectious virus in cough samples in 28% of the patients where the “control” NP or TS or saliva was positive for infectious virus, but not one continuous “speech” sample was found to have infectious virus and was consistent with the relatively high C_t_ values found in these specimens. These observations may be explained by the very high ratio of viral RNA-to-PFU that characterizes diverse clinical specimens (~ 160,000:1). A specimen would need to exhibit a relatively low C_t_ (≤ 25 in this study using N-gene probes) if a C_t_ was to be used as a surrogate for predicting the presence of infectious material. The presence of breath moisture condensate in our collection bags suggested we captured small aerosols adequately. Many studies have placed reliance on any C_t_ value, regardless of how high it was, as evidence that infectious material is also present in collected specimens but the actual C_t_ value and its correlation with a SARS-CoV-2 in vitro culture specimens is important. The public health implications of our findings would suggest that a short 5-min conversation with an infected COVID-19 patient at short distances in the absence of coughing or sneezing would be unlikely to be responsible for transmission of SARS-CoV-2 in most settings. We cannot rule out the possibility that shouting and/or singing^34^ would exhibit differing results. Difficulties associated with detecting viruses in air samples has been reported^34,35^.

The finding of a high quantitative burden of virus in cough and sputum samples would lend support to the transmission of SARS-CoV-2 in the large droplets that are typically generated by these events. Macroscopic droplets of saliva and/or respiratory secretions were readily observed in the collection bags in almost all cases. Our findings are congruent with a previous study which found that cough and sneeze samples in infected volunteers were found to have cultivatable Coxsackievirus A type 21 and the virus was carried in these droplets in an airflow dependent manner by large floor fans in an enclosed army barracks and infectious virus was detected in large air samplers on the side opposite to inoculated volunteers^36^.

Our observations suggest that kisses and human hands could be important for direct contact transmission given the high frequency of habitual human behaviours such as nose, lips and eye touching (up to 15.7 times per hour) and nose-picking (up to one of every three subjects^37,38^. This would allow inoculation of a relatively high virus burden directly onto sites bearing ACE2 receptors. This mode of transmission has previously been documented in the seminal studies when susceptible volunteers were asked to touch their nasal or conjunctival mucosa with fingers previously contaminated with a dried drop of Type 39 rhinovirus^38^ with reported attack rates of 36.4% in human challenge experiments.

SARS-CoV-2 also appears to be stable in clinical specimens in the context of the patient care setting. There are multiple in vitro experimental studies which have shown similar survival of SARS-CoV-2 on both porous and non-porous (e.g. plastic and polymer) surfaces over time frames ranging from several hours to four or more days^39–41^. Our data confirms the same virus stability using actual clinical specimens directly from patients and from their immediate environment in both the hospital and community settings and provides strong support for the fomite route of transmission.

An abundance of previous studies documents the transfer of infectious respiratory viruses from inanimate objects (fomites) that have been contaminated with nasal or respiratory secretions involving transfers onto the fingertips and then to the mucous membranes of the nose, mouth, and eyes. These investigations have employed human challenge, epidemiologic, virologic and intervention studies^42^. Attack rates of 50% and 56% were observed when recipients handled coffee cups and plastic tiles, respectively, that had been previously contaminated with a clinical strain of rhinovirus by infected donors^43^. Infectious rhinoviruses are also found on high-touch surfaces in home settings^44,45^. It has been reported that HCoVs OC43 and 229E exhibit a stability comparable to rhinovirus and are much more stable than influenza virus^42^. Other studies have shown that when preparations of SARS-CoV-1, MERS and other HCoV are suspended in a matrix resembling lung cell debris to mimic natural respiratory secretions, they can persist up to nine days on inanimate surfaces, further implicating this as a transmission mode^46–48^. A recent study on the survival of SARS-CoV-2 mixed with mucus from the upper respiratory tract revealed a survival time analysis of 10.2–12 h on human skin which corroborates our findings^49^. Infectious MERS was also isolated from fomites in a hospital setting including bedrails, bedsheets, an anteroom table and an IV hanger^50^. Our strong supportive evidence for fomite transmission does not align with other studies and commentaries^14,51,52^ which reported no infectious SARS-CoV-2 in the environment and discounted fomites as a risk for transmission. However, unless the sampling is done when patients are typically infectious, such studies would be highly compromised to detect any SARS-CoV-2.

Our findings provide unique insights into understanding the contagiousness of this virus. Some of the most infectious saliva and cough specimens exhibited virus loads approaching 10^6^ PFU/mL, suggesting that 10–100 µL droplets could deposit 10^4^–10^5^ PFU of infectious material. This is comparable to the titers of virus that can be obtained through cell culture in a controlled laboratory setting. Respiratory tract specimens were used as a typical “fomite” seed and the fact that purified clones of SARS-CoV-2 derived from this source caused infection in the Syrian hamster model provides direct evidence of the transmissibility of the virus to another mammalian host. It thus fulfills both Koch’s postulates and the Gwaltney-Hendley postulates of viral causation^45,53^. More critically we could produce disease with doses of only 14–30 PFU with these isolates and the minimum infectious dose in Syrian hamsters has been reported to be as low as 1 TCID_50_ with other stocks^25^. It is quite conceivable that the minimal infectious dose in humans is in the range of 1–5 PFU which is extraordinarily low. Experiments in human challenge studies in the Common Cold Unit from the 1960s showed that as little as 10^0.6–1.5^ TCID_50_ (~ 3–20 PFU) of a HCoV could cause infection with attack rates ranging from 17 to 67% of inoculated volunteers^12^. Thus, very few particles^54^ of a relatively stable virus may be capable of transmitting SARS-CoV-2 between humans, which would favour multiple routes of transmission and contribute to its relative contagiousness.

Our study has several strengths including the large number of patients recruited and the ability to carefully establish the timing of symptom onset through a detailed chart and record review of the medical interviews by experienced healthcare providers. We could detect, titer and identify infectious virus with relative ease in many samples from a diverse group of patients in both the hospital and community setting, including immunocompromised hosts. This was made possible by using a sampling-enriched strategy that focused on patients with a low C_t_ value. We made a purposeful decision to recruit patients in the early stages of symptomatic illness allowing optimal use of the labour-intense resources to quantitatively culture hundreds of specimens which we felt added strength of association to our hypothesis. Although this approach may have potentially limited the scope of the findings with respect to demonstrating high C_t_ values and lack of cultivatable virus, there are other published findings which support this observation^18,20,21^.

We recognize our study has limitations. Although most large-plaque forming SARS-CoV-2 strains plate equally efficiently on Vero CCL-81 cells and on TMPRSS2 transduced cell lines, we did detect a couple of specimens that were more easily detected on the latter cells. By mostly using CCL-81 cells there may have been a few other missed isolates. We were unable to collect serial specimens in many cases. Our continuous speech sampling strategy was limited by the health of the patients, and we cannot rule out that more prolonged acquisition times or singing or airflow dependent droplet carriage might detect infectious virus. We also have limited numbers of experiments on the effects of drying on the virus infectiousness and most are relegated to the hospital environment. Similarly, the hand transfer experiment was not done in replicate and there remains the possibility that there was infectious virus on the cleansed hand as no cultures were obtained before the handshake to document no pre-existing virus. However, shear forces created by the friction of the cleaning and previous studies demonstrating over a 1–2 log reduction of virus with simple hand washing with water would argue against disregarding this finding^55^. We also recognize that the small number of kiss and hand specimens represents a limitation regarding generalizability but nonetheless the finding of infectious virus from these specimens is significant given the relative hardiness of this virus and its potential for transmission from these sources.

We have acquired a large collection of samples in a SARS-CoV-2 infected population and have sought to systematically quantify the actual burden of infectious virus in clinical and environmental samples, including fomites and our evidence is compelling that contact transmission is an important and overlooked mode of transmission. Our study provides a quantitative numerical framework for evaluating the risk of encountering an infectious virus particle, given the relationships between C_t_, RNA copies/PFU, and days post-symptom onset. Our findings add novel and unique findings to the literature relating to the science of the transmission of this virus. With a probable very low minimal infectious dose in humans, our detailed observations and findings would support that SARS-CoV-2 exploits multiple modes of transmission and would suggest it is important not to focus on a singular mode of transmission. A broad array of mitigation strategies, including attention to fomite control, offers the greatest degree of protection from transmission.

## Methods

### Participants and setting

Patients who were admitted to one of four Calgary, Alberta (AB), Canada hospitals (Foothills Medical Centre, South Health Campus, Peter Lougheed Centre, and Rockyview General) and one Edmonton, AB hospital (Misericordia Community Hospital) between April 22, 2020, and March 31, 2021, and with a COVID-19 diagnosis confirmed by reverse transcriptase real time-polymerase chain reaction (RT-PCR), were approached to participate in the study. For community participants, the responsible Medical Officer of Health Alberta Health Services (AHS) Public Health (PH) in Calgary provided a list of people who had tested positive for COVID-19 confirmed by RT-PCR and were approached by telephone to participate in the study in their community setting. All participants provided informed consent for the use of any previous clinical samples completed for COVID-19 testing, collection of additional clinical or environmental samples, and other clinical information for research purposes. The study was approved by the University of Calgary Conjoint Research Ethics Board (REB20-0444) and was conducted in accordance with all local guidelines and the principles of the Declaration of Helsinki. Details of the infected cases were collected by health professionals who were a part of the investigative team and included demographic background, medical history, symptom assessment, date of symptom onset, date of COVID-19 testing, and disease course. Depending on whether the individual was in the community or the hospital, the data was gathered from personal interviews or from a review of AHS inpatient records and/or PH community contact tracer reports (Epidemiologic Summary Reports). A comprehensive assessment tool covering symptoms and signs (new or worsening) associated with COVID-19 was used for all inpatient cases, including core respiratory and gastrointestinal symptoms and signs and a COVID-19 expanded list including headache, muscle/joint pain, fatigue/extreme exhaustion, nausea/sudden loss of appetite, conjunctivitis/red eye/conjunctival edema, loss of/change to sense of smell or taste and any additional COVID-19 symptoms at the clinician’s discretion (e.g. cutaneous manifestations such as “COVID toes”). This assessment was supplemented by review of medical inpatient records and assessment by an innovative electronic clinical decision support COVID-19 symptom monitoring tool administered up to three-times daily. Serial follow up was used as required for outpatients, to improve sensitivity and reduce anchoring, recall, selection and inadequate follow up bias^31^. Patients were selected based on a range of early and late symptom onset and C_t_ values. Soon after the study was initiated it was recognized that patients with low C_t_ values (≤ 25) were more likely to produce cultivatable virus and these cases were preferentially enrolled.

### Clinical samples and sample collection

Clinical specimens and environmental samples were collected and tested using RT-PCR^56^ and quantitative SARS-CoV-2 plaque assays^57^. Clinical specimens included nasopharyngeal swabs (NP), throat swabs (TS), saliva, endotracheal aspirates (ETT), sputum if available, cough samples (spontaneous or requested), continuous speech, and clinical tissue samples and blood if appropriate. NP were collected using Flexible Mini-tip FloqSwabs (Copan) and TS using Puritan polyester or Copan ClassiqSwabs. Saliva (~ 1 mL) or ETT (1 to 2 mL) were collected into sterile containers. For each of these specimens, 3 mL of Copan UTM-RT or Dulbecco's Modified Eagle’s Medium (Gibco) with addition of 2% fetal bovine serum, 1 µg/mL meropenem and 1 µg/mL Amphotericin B (DMEM+) was added.

Methodologies for sample collections from the patient environment were assessed for ease and feasibility in the first few patients. Environmental samples were collected in a similar manner using an NP (Flexible Mini-tip FloqSwab, Copan), a sterile polyester -tipped throat swab, a sterile 2 × 2 cm cotton gauze, small pledgets cut from contaminated cloth samples (e.g., facecloth), or facial tissues to which 2 to 8 mL of DMEM+ was added. Cough and continuous speech samples were collected in sealable, transparent polyethylene bags (~ 27 × 27 cm and 18 × 19 cm, respectively) which were opened wide enough to accommodate the mouth and lower face and held in place at a distance < 2–4 cm to ensure adequate sample collection. Two to 5 mL of DMEM + was added to the bags, followed by thorough mixing to ensure the contents of the bags, including the condensation on the inside surfaces from the continuous speech samples and retained air would have contact with the culture media, prior to closing and sealing, given some air was expelled with sealing. Samples were processed in a biosafety cabinet for retrieval of the DMEM+ in preparation for transport to the Biosafety Level 3 (BSL3) containment facilities. All cough samples were accepted regardless of the vigour of the cough or if sputum was produced or not. The speech samples were continuous and directly observed by experienced collecting investigators (JC or TL) over 3–5 min and only accepted if no coughing, sneezing, or saliva contamination were observed. The kiss samples were obtained by a lip touch to the inside of the polyethylene bag and were done to simulate the double to triple kisses to the cheeks that are commonly used as a greeting in many cultures around the world. Random samplings of the polyethylene bags were cultured to ensure they were devoid of contaminating bacterial and fungal microbes that would interfere with plaque assays. Hand samples were collected by washing each hand in 10 mL of DMEM + for ~ 20 to 30 s in large 27 × 27 cm transparent, sealable polyethylene bags. Facial tissue samples (discarded post nose blowing) were collected and placed in 5 mL of DMEM + . Cell phone, call bell and nasal prong samples were collected using a 2 × 2 cm sterile cotton gauze which was added into 3 to 7 mL of DMEM+. Denture samples were collected by adding a few drops of saline to the denture groove, swabbing the area, and then placing the swab into 1 mL DMEM + or by using a dry swab as noted above to which 1 mL DMEM+ was added.

### Effects of drying on clinical samples of saliva with infectious SARS-CoV-2

Saliva samples were collected into sterile containers and 1–2 mL aliquots were placed into sterile polystyrene Petri dishes to study the effects of desiccation and to assess virus viability. Each sample was either neat or mixed with 6 mL DMEM+ and the latter alone was also used as a negative media control. The baseline sample was stored in a sealed container at room temperature throughout the experimental period after which DMEM+ was similarly added. The Petri dishes containing saliva samples were left open to the air in the patient care room for 2 h to enable significant drying which was visually confirmed. The dried samples were then resuspended in 2 mL of DMEM+ and transferred to a sterile collection tube.

### Hand transfer of infectious SARS-CoV-2

A COVID-19 patient, with an initial C_t_ value of 14.7 and 16.9 (targets 1 and 2) on the Xpert Xpress SARS-CoV-2 test (Cepheid) was one day post symptom onset and had cough as one of the symptoms. The patient’s left hand was vigorously cleaned by the investigator with wet paper towels, ensuring friction was applied to all surfaces. The patient was asked to cough on their right hand and then to shake their left hand within ~ 20 s. The handshake was a few seconds in duration. Hand samples were collected post handshake from each hand as described above.

### Sample transport

All the freshly collected samples were placed on ice packs within 1-to-4 h and then refrigerated at 4 °C for up to 48 h in a secure location before being transported at 0–4 °C to Edmonton, AB. If the samples could not be transported immediately, they were flash-frozen in a dry-ice ethanol bath and then forwarded on dry ice. Upon arrival in Edmonton, the samples were transferred to the BSL3 containment facility and assayed for infectious virus within 24-to-48 h of their collection. If that were not possible, a few samples were snap frozen and stored at − 80 °C until they could be titered.

### Cell culture and virus titration

Vero (ATCC #CCL-81) and Vero E6/TMPRSS2 (JCRB cell bank 1819) were cultured with Modified Eagle’s Medium (MEM, Gibco) supplemented with 100 units/mL of penicillin, 100 µg/mL of streptomycin, 0.25 µg/mL of Amphotericin B (Gibco), and 10% fetal bovine serum (Gibco). For virus culture, 2 × 10^5^ cells were seeded into each well of the 12-well plates one day before titering. Ten-fold serial dilutions of the virus were plated in duplicate on Vero CCL-81 cells and cultured for 3 days at 37 °C in MEM supplemented with 100 units/mL of penicillin, 100 µg/mL of streptomycin, 0.25 µg/mL of Amphotericin B, and 1% carboxymethyl cellulose (CMC) (Sigma). In a few cases, detected late in the study, some slow-growing viruses that produced small plaques on Vero CCL-81 cells were also plated on Vero E6/TMPRSS2 cells^22^. The cells were then fixed and stained with a solution containing 0.13% (w/v) crystal violet, 11% formaldehyde (v/v), and 5% ethanol (v/v) to permit plaque counts. All sample processing in the BSL3 laboratory was conducted with the authorization of the University of Alberta’s Human Research Ethics Board (Pro00099761) and Office of Environmental Health and Safety (RES0052249).

### Confirmation of SARS-CoV-2 from clinical specimens

A SARS-CoV-2 strain (GISAID# EPI_ISL_425177) was received from the Vaccine and Infectious Disease Organization at the University of Saskatchewan and used as a positive control and plaque reference. Like the clinical isolates described in this report, this strain formed plaques that exhibited a halo-like appearance on Vero CCL-81 cells under a CMC overlay. To further confirm the identity of a subset of virus isolates, the fixed and strained plates were de-stained with ethanol and then immunostained with a 1:500 diluted rabbit anti-SARS-CoV-2 spike antibody (ProSci). The plaques were then visualized with a 2° goat anti-rabbit IgG antibody conjugated to horseradish peroxidase (Invitrogen) and a KPL TrueBlue peroxidase substrate (SeraCare).

### RT-PCR assays and viral sequencing

E gene reverse transcriptase real-time PCR (RT-PCR) for SARS-CoV-2 was performed as described by^58^. Samples were considered positive when E gene C_t_ value was < 35. If the C_t_ was ≥ 35, amplification from the same eluate was repeated in duplicate and was considered positive if at least 2/3 results had a C_t_ < 41. For N gene RT-qPCR, viral RNAs were extracted using QIAamp viral RNA min kit (Qiagen). RT-qPCR was performed using US CDC SARS-CoV-2 Research Use Only qPCR Primer and Probe Kit (Integrated DNA Technologies; 2019-nCoV RUO Kit), GoTaq 1-step RT-qPCR system (Promega), and Bio-Rad CFX96. All the RT-qPCR analyses conducted using N2 primer sets were performed in parallel with control wells containing known quantities of an N-gene DNA target (2019-nCoV_N_Positive Control; Integrated DNA Technologies) which permitted a conversion of C_t_ values to molecular quantities (SQ) (supplemental data). For the current analysis, the study relied primarily upon N2-based molecular detection and quantitation. Any other samples submitted for diagnostic testing were done using Health Canada/FDA approved tests at a laboratory accredited by the College of Physicians and Surgeons of Alberta.

Amplicon-based enrichment of SARS-CoV-2 was carried out using a QiaSeq Direct SARS-CoV-2 library preparation kit (Qiagen). Briefly, viral RNA was reverse transcribed into cDNA, followed by a high-fidelity multiplex PCR reaction using two pools of overlapping SARS-COV-2-specific 250 bp amplicons that span the length of the genome. Following the addition of unique dual indices to each sample, the libraries were sequenced using an Illumina MiSeq v2 (300 cycle) kit, generating an average of 2 million reads per sample. The quality control, generation of libraries and sequencing run were all performed at The Applied Genomics Centre in the Faculty of Medicine and Dentistry at the University of Alberta.

Bioinformatics analysis was performed with CLC Genomics Workbench v21 using the “Identify Qiaseq SARS-CoV-2 low frequency and shared variants (Illumina)” workflow. Paired-end trimmed reads were mapped to the SARS-CoV-2 genome (Genbank MN908947.3). The alignment was refined using the InDels and Structural Variants module, followed by the local realignment module. Nucleotide variants were identified by a minimum coverage of five reads and a minimum frequency of 70%. Consensus sequences were generated and submitted to the Pangolin lineage assigner (https://pangolin.cog-uk.io) to determine SARS-CoV-2 lineages.

### Statistical analysis

Statistical analysis was performed using GraphPad Prism v9 with additional calculations performed with Microsoft Excel v16.50 and appropriate tests of significance applied for continuous or dichotomous variables. The Pearson correlation coefficient was used as a measure of linear correlation between two sets of data. An unpaired *t*-test, using Welch’s correction for unequal variances and sample sizes was used to compare C_t_ for infectious versus non-infectious samples. In all cases, a *p* value < 0.05 was considered significant. Please see the figure legends for specific details.

### Stability of SARS-CoV-2 on surfaces, medical equipment and N95 respirators

We inoculated several types of commonly used pieces of medical equipment that are often used between patients including: stethoscope diaphragm, pulse oximeter, a bedside call bell, a keyboard, and a small personal digital device cover with clinical samples of saliva and ETT secretions known to be culture positive for SARS-CoV-2. The common touch surfaces of the equipment (e.g., diaphragm of the stethoscope, inside portion of the pulse oximeter placed on the finger) were inoculated to determine the effects of desiccation on the titer of the virus over time and to mimic bedside settings where these pieces of equipment might be exposed to saliva or sputum during the course of a patient’s COVID-19. We placed 10 µL of saliva and endotracheal secretions collected from patients at titers of 2.5 × 10^4^ and 1 × 10^6^ PFU/mL, respectively, to each piece of equipment. We allowed the inoculum to dry for periods of 30, 60 and 240 min to mimic actual clinical scenario environments including the time to conduct an interview and complete a physical exam by a bedside clinician and a 4-h time interval for a check by nursing staff during a nighttime shift. The inoculated pieces were reconstituted with 400 µL of DMEM + and any recovered virus was quantified by plaque assay on Vero cells. We also inoculated 10 µL of a clinical sputum specimen with a titer of 1.2 × 10^5^ PFU/mL obtained from a COVID-19 patient onto coupons cut under sterile conditions from N95 respirators (Halyard Fluidshield 46,727 duckbill respirator, 3 M 1860 half-sphere respirator, and 3 M 1870 + panel respirator)^59^. As controls (0 min), virus inoculants were applied to the surfaces of the medical instrument cut-outs and immediately those cut-out segments were placed into SF MEM to eluate viruses. The outer surface of the N95 respirator was inoculated to determine the effects of desiccation on the titer of the virus over time to mimic a bedside setting where a SARS-CoV-2 infected patient may cough or sneeze and deposit saliva or sputum on the outer surface of a mask. We dried the coupons for 30 min and then eluted and quantified the virus by plaque assay as noted above.

### Transmission and virulence studies in a Syrian hamster model

Eight-week-old Syrian male hamsters (*Mesocricetus auratus*) were purchased from Charles River Laboratories, Montreal, Quebec. Hamsters are highly susceptible to SARS-CoV-2^25^ and all of the studies were conducted in BSL3 containment with the approval of the University of Alberta’s Animal Care and Use Committee under authorization AUP00001847. The studies used two different lineages (B.1.279 [EPI_ISL_3526025] and B.1.128 [EPI_ISL_3526026]), isolated from COVID-19 patient respiratory tract samples, plaque purified three times and expanded once by passage on Vero CCL-81 cells. The hamsters were anesthetized with isofluorane, infected intranasally with 100 µL of SARS-CoV-2 (50 µL/nare) and returned to their cages. The animals were subsequently weighed daily as well as being swabbed on days 1, 3, and 6 within the nose and on the mouth and tongue with a polyester swab (Puritan). The swabs were placed in 600 µL of Iscove’s modified Dulbecco’s medium containing 2% fetal calf serum and stored frozen at − 80°. The hamsters were euthanized on day 14 and the four smaller lobes of the lungs homogenized in 2 mL of MEM using a GentleMacs M tube, followed by centrifugation for 10 min at 3000×*g* and 2 min at 8000×*g*. The supernatants were aliquoted with a portion reserved for RNA extraction and another for virus titration.

Virus culture and titration was conducted as described above. For PCR quantification, 140 µL of virus-containing sample was first mixed with 0.56 mL of “AVL” viral lysis buffer and carrier RNA and processed to extract the RNA using a QIAamp viral RNA minikit (Qiagen). Five microliters of RNA extracted from the oral-nasal swabs, or 5 µL of 1:10 diluted RNA from the lung homogenates, were then analyzed using a Promega Go-Taq One-step RT-qPCR kit and CDC nucleocapsid primer set and cycling protocol (IDT) as described above.

## Supplementary Information

Supplementary Information.

## Data Availability

Genome sequence data for clinical isolates 56B and 72B were uploaded to GISAID with the following numbers EPI_ISL_3526025 and EPI_ISL_3526026. All other raw data available upon request.
