# Supplementary material for: Detection and quantification of infectious severe acute respiratory coronavirus-2 in diverse clinical and environmental samples
Source: Sci Rep. 2022 Mar 30;12:5418. doi: 10.1038/s41598-022-09218-5 (PMC8967087; doi:10.1038/s41598-022-09218-5)
Supplement: Supplementary file 1 — Supplementary Information. [file 41598_2022_9218_MOESM1_ESM.docx]

**Supplemental File: Primers and Protocols E and N Genes**

**Table 1. RT-PCR primers and probes**

| **Target** | **Name** | **Sequence (5’-3’)** | **Source** |
| --- | --- | --- | --- |
| E gene | COVID19_E_For_V2 | GAGACAGGTACGTTAATAGTTAATAGCG | In house |
| E gene | COVID19_E_Rev_V2 | CAATATTGCAGCAGTACGCACAC | In house |
| E gene | COVID19_E_MGB_FAM | CTAGCCATCCTTACTGCG (FAM/MGB) | In house |
| N gene | 2019-nCoV_N1-F | GACCCCAAAATCAGCGAAAT | IDT |
| N gene | 2019-nCoV_N1-R | TCTGGTTACTGCCAGTTGAATCTG | IDT |
| N gene | 2019-nCoV_N1-P | FAM-ACCCCGCATTACGTTTGGTGACC-BHQ1 | IDT |
| N gene | 2019-nCoV_N2-F | TTACAAACATTGGCCGCAAA | IDT |
| N gene | 2019-nCoV_N2-R | GCGCGACATTCCGAAGAA | IDT |
| N gene | 2019-nCoV_N2-P | FAM-ACAATTTGCCCCCAGCGCTTCAG-BHQ1 | IDT |

**E gene RT-PCR protocol:**

The RT step was performed at 50°C for 5 minutes followed by incubation at 95°C for 20 seconds. Amplification included 45 cycles of denaturation at 95°C for 3 seconds, followed by annealing, extension, and data acquisition at 60°C for 30 seconds on the 7500 Fast RealTime PCR system (ABI).

**N gene RT-qPCR protocol:**

The RT step was performed at 45°C for 15 minutes followed by incubation at 95°C for 2 minutes. Amplification included 40 cycles of denaturation at 95°C for 3 seconds, followed by annealing, extension, and data acquisition at 55°C for 30 seconds on the Bio-Rad CFX96 real time PCR machine. Standard curves of a SARS-CoV-2 N positive control (IDT) was performed at starting concentration of 10^6^ copies/well with a series of 10-fold dilutions, in parallel with each sample run.
